# What’s new in multidrug-resistant pathogens in the ICU?

**DOI:** 10.1186/s13613-016-0199-4

**Published:** 2016-10-06

**Authors:** Gabor Zilahi, Antonio Artigas, Ignacio Martin-Loeches

**Affiliations:** 1 Department of Clinical Medicine, Trinity Centre for Health Sciences, Multidisciplinary Intensive Care Research Organization (MICRO), Wellcome Trust‐HRB Clinical Research, St James’s Hospital, St James’s University Hospital, Dublin 8, Ireland; 2 Critical Care Center, Parc Taulí Hospital-Sabadell, CIBERes, Parc Tauli s/n., Sabadell, Barcelona, Spain; 3 Centros de Investigación Biomédica en Red (CIBER), Madrid, Spain; 4 Wellcome Trust‐HRB Clinical Research, Dublin, Ireland; 5 Department of Clinical Medicine, Trinity Centre for Health Sciences, Dublin, Ireland

## Abstract

Over the last several decades, antibacterial drug use has become widespread with their misuse being an ever-increasing phenomenon. Consequently, antibacterial drugs have become less effective or even ineffective, resulting in a global health security emergency. The prevalence of multidrug-resistant organisms (MDROs) varies widely among regions and countries. The primary aim of antibiotic stewardship programs is to supervise the three most influential factors contributing to the development and transmission of MDROs, namely: (1) appropriate antibiotic prescribing; (2) early detection and prevention of cross-colonization of MDROs; and (3) elimination of reservoirs. In the future, it is expected that a number of countries will experience a rise in MDROs. These infections will be associated with a high consumption of healthcare resources manifested by a prolonged hospital stay and high mortality. As a counteractive strategy, minimization of broad-spectrum antibiotic use and prompt antibiotic administration will aid in reduction of antibiotic resistance. Innovative management approaches include development and implementation of rapid diagnostic tests that will help in both shortening the duration of therapy and allowing early targeted therapy. The institution of more accessible therapeutic drug monitoring will help to optimize drug administration and support a patient-specific approach. Areas where further research is required are investigation into the heterogeneity of critically ill patients and the need for new antibacterial drug development.

## Background

International health organizations, including the European Centre for Disease Prevention and Control (ECDC) and the Centers for Disease Control and Prevention (CDC), have used terms such as “crisis,” “catastrophic consequences” and “nightmare scenario” to highlight the rapid emergence and spread of antibiotic resistance [1–4]. The World Health Organization (WHO) has identified antimicrobial resistance as one of the most important problems for human health with significant adverse impacts on clinical outcomes and higher costs due to consumption of healthcare resources [5].

## Epidemiology of highly resistant bacteria

A number of studies have been performed to assess the burden of infection in critical illness. The Intensive Care Over Nations (ICON) audit showed that more than one-third of the patients develop an infection during their intensive care unit (ICU) stay [6]. The Extended Prevalence of Infection in Intensive Care (EPIC) II study showed that 51 % of patients were considered to be infected while in ICU. The infection was of respiratory origin in 64 % of cases. *S aureus* (20.5 %) was the most frequent organism isolated, despite the overall predominance of Gram-negative organisms as a group: 62.2 % (*E. coli*, *Enterobacter s*pp., *Klebsiella* spp., *Pseudomonas* spp. and *Acinetobacter s*pp.) [7].

Antibiotic resistance is a serious problem in all parts of the world including Asia–Pacific, Latin America, Middle East, Europe and North America regions. A particular concern is the misuse or overuse of antibiotics, which has lead to the development of resistant or super-resistant bacterial strains. Two regions of particular concern, with existing high levels of antimicrobial resistance, are South-East Asia and the Middle East where antibiotics can be easily bought over the counter [8, 9]. Findings from a cross-sectional study from Saudi Arabia showed that antibiotics were dispensed without a medical prescription in 244 (77.6 %) of 327 pharmacies analyzed, of which 231 (95 %) were dispensed without a patient request [10].

In Europe, the European Antimicrobial Resistance Surveillance Network (EARS-Net) has provided European reference data on antimicrobial resistance for public health purposes since the program began in 1999. Over the last 4 years (2011–2014), the proportion of *K. pneumoniae* and *E. coli* with resistance to fluoroquinolones, third-generation cephalosporins, aminoglycosides and a combined resistance to all three antibiotic groups has increased significantly. Large inter-country variations in *Acinetobacter* spp. resistance exist in Europe with a high percentage of resistant isolates being found in southern and southeast Europe. Similar patterns are observed with methicillin-resistant Staphylococcus aureus (MRSA) with resistance levels varying from 0.9 to 56 % depending on the country studied [11]. The recent “European Antimicrobial Resistance One Health ministerial conference 2016” highlighted the substantial antimicrobial resistance problem in Europe. For several antimicrobial group–bacterium combinations, a north-to-south and west-to-east gradient is evident in Europe. In general, lower resistance percentages are reported by countries in the north and higher percentages by countries in the south and east of Europe [12].

**Fig. 1:**
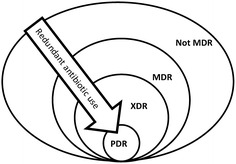
Multidrug-resistant organisms (MDR) have been divided into three categories depending on their resistance profile: 1. MDR—non-susceptible to at least 1 agent in 3 antimicrobial categories; 2. extensively drug-resistant (XDR)—non-susceptible to at least 1 agent in all but 2 or fewer antimicrobial categories and 3. pan-drug-resistant (PDR)—non-susceptible to all agents in all antimicrobial categories

A group of international experts brought together by a joint initiative between the ECDC and the CDC, was tasked with creating a standardized international terminology to describe acquired resistance profiles in multidrug-resistant organisms [13]. MDROs have been divided into three categories depending on their resistance profile: 1. MDROs—non-susceptible to at least 1 agent in 3 antimicrobial categories; 2. extensively drug-resistant (XDR) organisms—non-susceptible to at least 1 agent in all but 2 or fewer antimicrobial categories; and 3. pan-drug-resistant (PDR) organisms—non-susceptible to all agents in all antimicrobial categories (Fig. 1).

Over recent years, a new, comprehensive recommendation on classification of infections caused by Gram-positive and emerging Gram-negative multidrug-resistant pathogens has been launched. *E. faecium, S. aureus, K. pneumoniae, A. baumannii, P. aeruginosa* and *Enterobacter* spp. (ESKAPE) pathogens account for more than 80 % of infectious episodes in the ICU. As this acronym seems to help to highlight the problem of MDROs, some authors [14] claim a change to “ESCAPE” is warranted (*E. faecium, S. aureus, C. difficile, A. baumannii, P. aeruginosa* and *Enterobacteriaceae* spp.) in order to highlight the importance of *C.* difficile and incorporate not only *Enterobacter* spp. but also other *Enterobacteriaceae*spp. (namely, *Escherichia coli and Proteus* spp.) because of the increasing levels of antibiotic resistance (including extended-spectrum β-lactamases, carbapenemases and aminoglycoside resistance) and decreasing levels of fluoroquinolone susceptibility among these organisms*. E. coli* infections currently account for ~20 % of all cases of bacteremia in the UK and Ireland [15] with not just high rates of fluoroquinolone and amoxicillin–clavulanic acid (AMC) resistance, but also extended-spectrum beta-lactamase (ESBL) mechanisms with a possible link between high resistance and community antibiotic misuse [16]. In addition, ESBL *Enterobacteriaceae* spp. are responsible for outbreaks irrespective of location. Antibiotic selection of ESBL-producing Gram-negative pathogens is not just related to the total use of antimicrobial agents in hospitals, but also to the particular use of the fluoroquinolones and second- and third-generation cephalosporins [16]. *P. aeruginosa* has an extensive capability to become resistant to all type of antibiotics, create biofilms and demonstrate a high level of intrinsic resistance. These characteristics are caused by production of ESBL, overexpression of AmpC beta-lactamases and other drug-resistant mechanisms such as production of metallo-β-lactamases (MBLs) [17]. *Acinetobacter* spp., similar to *P. aeruginosa*, have an array of mechanisms by which they develop and horizontally transfer resistance. The most important of these is the production of beta-lactamases and aminoglycoside-modifying enzymes [18].

**Fig. 2:**
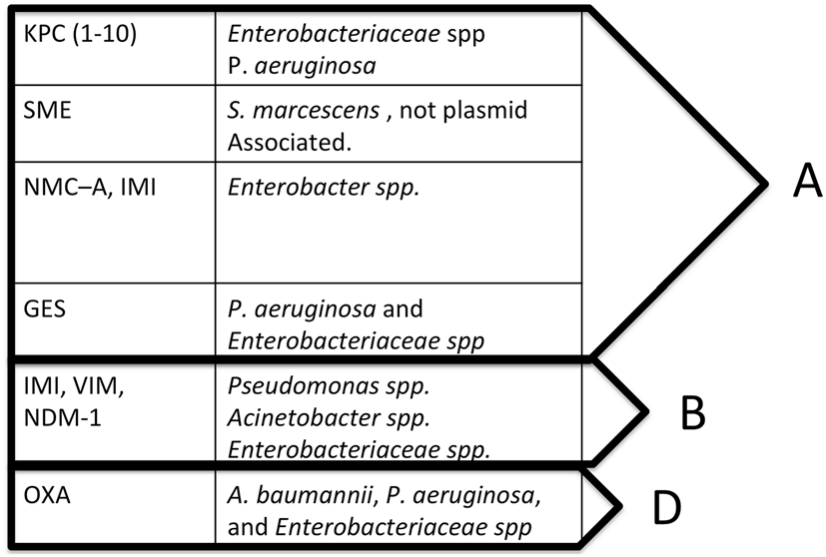
Types of carbapenemases. *KPC Klebsiella pneumoniae* carbapenemase, *SME Serratia marcescens* enzyme, *MNC* not metallo-carbapenemase, *IMI* imipenem-hydrolyzing β-lactamase, *GES* Guiana extended spectrum, *IMI* imipenem-hydrolyzing β-lactamase, *VIM* Verona integron-encoded MBL, *NDM*-*1* New Delhi metallo-β-lactamase

As a result of misuse of antibiotics, different pathogens are becoming increasingly resistant. The lack of regulatory policies regarding antibiotic prescription makes them easily accessible and cheap, which promotes overuse [19]. Gram-negative bacilli are often resistant to multiple antimicrobials. It is important to mention the newly emergence of the first plasmid-mediated polymyxin resistance mechanism, MCR-1, in Enterobacteriaceae in China [20]. This is especially worrisome because plasmid-mediated resistance can spread in an explosive manner and de-activate polymyxins, which are one of our last antibiotic options. On the other hand, carbapenems are another important last-line group of antibiotics for infections involving multidrug-resistant Gram-negative *Enterobacteriaceae* spp. The most important mechanism of carbapenem resistance in this group is the production of carbapenemases, although resistance can also result from the synergistic activity between AmpC-type and extended-spectrum beta-lactamases combined with decreased outer membrane permeability. Three major molecular classes of carbapenemases are recognized: A, B and D (Fig. 2). Although carbapenem resistance remains at relatively low levels in northern Europe, it does constitute a serious threat worldwide as a consequence of acquisition of carbapenemase genes with a higher prevalence in southern Europe and Asia. In South America, carbapenem-resistant *P. aeruginosa* and *A. baumannii* could be as high as 56 and 35 %, respectively [21]. Outbreaks of class A carbapenemases particularly plasmid encoded (*K. pneumoniae* carbapenemases [KPC]) producers also have been reported in several countries (Greece, Israel, China, Brazil, Argentina and USA) [22]. Some authors have proposed two concomitant epidemics of carbapenemase producers worldwide [23]: (1) community-acquired infections with carbapenemase-producing *E. coli* (New Delhi metallo-β-lactamase-1) (NDM-1 and OXA-48 types) and (2) nosocomial carbapenemase producers in *K. pneumoniae* of all types (KPC, IMP, VIM, NDM and OXA-48).

There is an urgent need for development of new agents with activity against MDROs. The Infectious Diseases Society of America (IDSA) supported a program, so-called Bad Bugs, Need Drugs; 10 × ‘20 Initiative, with the aim of developing ten new systemic antibacterial drugs by 2020 [24]. In Europe, a recent report from the Economist Jim O’Neill in the UK expressed concern that the global financial cost of no action would be the loss of 10 million lives a year by 2050 and £69 tn ($100 tn) a year [25]. Infections by MDROs were associated with worse clinical outcomes compared with their susceptible counterparts [26]. While higher mortality associated with MDROs may be related to the virulence of the pathogen and a weak (comorbidity, age, etc.) or severely ill host (organ failure status, etc.) [27, 28], it is also important to highlight that delays in effective antimicrobial administration are also associated with high mortality [29].

## Risk scoring/risk factors

There have been multiple attempts to identify patients at risk of MDROs with the implementation of risk scoring systems to initiate appropriate targeted antibiotic therapy. Unfortunately, the value of these scores is limited [30]. Most of the risk factors are shared among different MDRO species, and the same type of organism with various degrees of resistance might have distinct epidemiological characteristics. Martin-Loeches et al. [31] confirmed in a multicenter study that patients without risk factors listed in the 2005 ATS/IDSA guidelines admitted to ICU with hospital-acquired pneumonia (HAP) and/or ventilator-associated pneumonia (VAP) had a high prevalence of MDROs (50.7 %) confirming the difficulty of predicting MDROs based on risk stratification. Most clinicians use certain risk profiles (complex ICU stay, recent prior hospitalization or long-term care facility habitant, recent exposure to antimicrobials, etc.) to assess the need for broad-spectrum antibiotic therapy. This approach certainly improves adequate initial antibiotic choice, but again shares the same concerns with risk scores being non-specific and broad. If broad-spectrum antibiotics, based on risk scores or risk stratification, are prescribed, strict antibiotic stewardship programs should be also applied. This strategy, mainly based on de-escalation policies, will ultimately promote higher rates of appropriate antibiotic use. The recent launch of the IDSA/ATS guidelines for HAP/VAP will certainly help how to consider antibiotic treatment regarding the aforementioned concerns [32]. Strengths from these recently published guidelines are that VAP by MDR pathogens is significantly simplified and for the first time clinical parameters are included, namely septic shock on appearance of VAP and acute respiratory distress syndrome (ARDS) preceding VAP diagnosis.

Another approach is to perform regular surveillance. Based on previous observational studies MDROs colonization may precede bacteraemia in the ICU [33]. This early identification of MDROs—even before clinical infection—can guide the clinician toward appropriate empiric treatment. Brusselaers et al. [34] in a recent meta-analysis of tracheal aspirate surveillance concluded that such an approach has a high negative predictive value, i.e., if a recent surveillance culture does not contain MDROs, the newly symptomatic VAP is unlikely to be caused by MDROs (likelihood <10 %). However, the presence of MDROs only had a moderate positive predictive value with an overall 75 % sensitivity.

## Rapid methods to diagnose multidrug resistance

One of the key aspects of avoiding the spread of resistant strains is early detection with the use rapid diagnostic tests. The methods currently used in hospitals for microbial detection tend to be labor-intensive, consume human resources and take more than a day to yield results. Newly developed rapid microbial detection tests, which biopharmaceutical industries have been slow to embrace, are precise, sensitive and generate results faster when compared with traditional culture-plate methods. There has been growing experience with rapid diagnostic techniques within the last decade, especially with MRSA. Harbarth et al. [35] using quick, multiplex immunocapture-coupled PCR (qMRSA) were able to decrease median time to notification from four days to one day which can help to identify previously unknown MRSA carriers rapidly. Experience with Gram-negative isolates such as carbapenemase-producing organisms (CROs), including the *Enterobacteriaceae* spp., is also evolving. Detection of patients carrying carbapenemase-producing microorganisms can significantly impact on infection control. Recently, Tato et al. assessed the performance of the Cepheid Xpert Carba-R assay (designed for the rapid detection and differentiation of the blaKPC, blaNDM, blaVIM, blaOXA-48 and blaIMP-1 genes) and found that sensitivity, specificity, positive and negative predictive values compared to reference culture and sequencing results were above 95 %. Worryingly, in addition to CROs, there has been an increase in reports of *Enterobacteriaceae* spp. resistant to polymyxins. Since conventional methods for detection of colistin-resistant isolates such as microdilution are time-consuming and methods like disk diffusion and the E-test are not reliable, Nordmann et al. [36] evaluated a new test: a chromogenic agar, shortening the results by 16 h compared to the reference broth dilution method, with a sensitivity and specificity of 99.3 and 95.4 %, respectively, compared to the standard microdilution method.

Shortening the turn-around time of positive blood culture identification and susceptibility results is essential to optimize antimicrobial treatment in patients with severe infections. One of the most widely used technologies nowadays that actually has revolutionized the process of care in microbiology laboratories is the matrix-assisted laser desorption ionization/time-of-flight mass spectrometry (MALDI-TOF MS) [37]. The introduction of mass spectrometry through MALDI-TOF in the diagnosis of bacteraemia and fungemia has represented a revolution due to the rapidity and reliability of the results that it can offer to microbiology services and laboratories through analysis of the mass spectrum of the bacterial protein directly from positive blood culture bottles [38]. Management of patients with bacteraemia has been optimized with the introduction of this methodology due to the rapid information.

Rapid diagnostic tests implemented in the current clinical practice should also consider an adequate workflow of information within a multidisciplinary working group approach that can process quickly and correctly the results from the microbiology laboratory to the bedside of the patient. An adequate process of care performance of rapid diagnostic tests such as MALDI-TOF will improve the quality of care, with the reduction of antibiotic expenditure and hospital stay and helping to infection control with the implementation of adequate and timing transmission-based precautions [39].

The basis for all infection control measures is the accurate and timely laboratory identification of MDROs. This will also deliver important information for regional and national containment strategies and hospitals. The implementation of rapid tests and the development of microbiology laboratory reference services is an urgent requirement.

## Antibiotic stewardship (AMS) and infection control strategies

Antimicrobial stewardship (AMS) programs aim to provide assistance with optimal choice, dosage, pharmacokinetic–pharmacodynamic (PK/PD) characteristics and duration of antibiotics in order to reduce costs, adverse events and the development of resistance [40]. Even though the IDSA has published guidelines [41] about AMS programs, the ideal care bundle has yet to be identified as published articles vary greatly in their findings. A systematic review by Kaki et al. [42] assessing 24 mainly uncontrolled, before and after studies published in 2011 showed that marked heterogeneity exists with respect to interventions evaluated and no mortality benefit could be identified. Nevertheless, AMS was associated with an overall clinical benefit as evidenced by a reduction in antibiotic consumption, costs and antibiotic pressure that subsequently results in a decrease in resistance rates [42].

These measures are only effective if all stakeholders, supported by the relevant authorities, accept implementation and incorporate audits and feedback mechanisms. A recent example of such an initiative is the “Zero Resistance” program developed by The Spanish Society of Intensive Care Medicine and Coronary Care Units (SEMICYUC). The main objective of the “Zero Resistance” project is reduction in the cumulative incidence of patients with ICU-acquired MDR infections by 20 % with a bundle of 10 recommendations. The primary aim of the antibiotic program is to supervise the three most influential contributing factors to the development and transmission of MDROs, namely: (1) adequate prescription of antibiotics; (2) early detection and prevention of cross-colonization of MDR; and (3) elimination of reservoirs [43]. Results of this ambitious project will shed light on AMS programs in the near future.

De-escalation is defined as an AMS strategy of reducing the number of antibiotics or using narrower/targeted spectrum drugs. De-escalation has been found to be safe and associated with less antibiotic usage and shorter duration of therapy in observational studies. Theoretically, this will translate into a reduction of MDROs; nonetheless, it has yet to be clearly proven [44]. Two studies have recently been published looking at de-escalation in patients with severe sepsis. Leone et al. [45] conducted a multicenter non-blinded randomized non-inferiority trial of patients with severe sepsis and Garnacho-Montero et al. [46] a prospective observational study enrolling patients admitted to the ICU with severe sepsis or septic shock. While Leone et al. [45] found that de-escalation was inferior to continuation of the initial antibiotic therapy (length of stay as the primary outcome parameter), the study had important limitations (small numbers of patients and unbalanced groups at baseline; patients were younger and had lower disease severity). Garnacho-Montero et al. [46] demonstrated a protective effect of de-escalation in terms of mortality. In an observational, single-center study, Montravers et al. [47] prospectively analyzed the characteristics and outcomes of anti-infective de-escalation during healthcare-associated intra-abdominal infections and found that the presence of non-fermenting Gram-negative bacilli (NFGNB) and MDROs limited the implementation of a de-escalation program. De-escalation is a feasible option in patients with polymicrobial infections such as healthcare-associated intra-abdominal infections, but MDROs and NFGNB limit its implementation. Recently, De Bus [48] et al. analyzed the effect of de-escalation in 478 anti-pseudomonal antibiotic prescriptions and could not find lower rates of resistant organisms after exposure to anti-pseudomonal beta-lactam antibiotics. Interestingly, Carlier et al. [49] conducted a simulation study showing that the probability of achieving a PK/PD target was lower in the narrower-spectrum antibiotic group (amoxicillin–clavulanic acid, cefuroxime, flucloxacillin, cefazolin and cefepime) using conventional dosing as compared to broad-spectrum antibiotics like meropenem and piperacillin/tazobactam.

Combination antimicrobial therapy exploits the synergistic effect of some antibiotic groupings in addition to broadening the spectrum of activity against a suspected pathogen to ensure adequate coverage. The synergistic effect of combination therapy has been shown to be beneficial in vivo in invasive pneumococcal disease and toxic shock syndrome. Randomized controlled trials (RCTs), on the other hand, have failed to demonstrate a mortality benefit with B-lactam and aminoglycoside combinations, but renal damage was more frequent [50]. Treatment of infections caused by CROs using a combination of two or three active drugs has consistently been associated with improved outcomes, with carbapenems being included in the combination when the minimal inhibitory concentration (MIC) of the pathogen against meropenem does not exceed 8 mg/L [51, 52]. However, a survival benefit when using combination treatment for infections caused by multidrug-resistant Gram-negative bacteria is questionable [53–55]. As almost all these data originate from observational studies, the need for RCTs is clear.

There is an on-going debate about pharmacokinetic and pharmacodynamic (PK/PD) optimization. On the one hand, the critically ill patient has a higher than normal volume of distribution, impaired renal and hepatic clearance, altered plasma protein content and they also tend to be elderly with significant comorbidities. On the other hand, there is growing evidence that in selected patient populations renal clearance is actually augmented, which can lead to inadequate plasma concentrations for some of the important antibiotic groups like carbapenems, beta-lactams and cephalosporins. Yang et al. [56] published a systemic review showing that the extended or continuous infusion of beta-lactams (piperacillin/tazobactam) strategy was associated with a higher clinical cure rate and lower mortality than the conventional intermittent approach. In patients with multiorgan failure and renal replacement therapy, residual diuresis, type of membrane and body weight should be considered for beta-lactam dose titration [57, 58]. Another frequently used drug class to treat MDROs are the aminoglycosides. Recent studies have highlighted the need for dose adjustment in critically ill patients. De Montmollin et al. [59] found that one-third of the patients in ICU had an amikacin *C*_max_ < 60 mg/L with the recommended dose of 15 mg/kg of total body weight. This suggests the need for dose optimization based on creatinine clearance, total body weight and positive 24-h fluid balance in order to reach adequate therapeutic targets [60, 61]. Until drug assays are routinely available, a personalized approach to dosing should be considered for each patient based on local AMS.

Because of the increasing prevalence of Gram-negative MDROs worldwide, previously discarded antibiotics are being re-evaluated. Recent data suggest that the current dosage regimens of colistin are suboptimal in many critically ill patients [62]. To optimize the plasma level the administration of a loading dose has been proposed [63]. It has also recently been shown [64] that the dose of other “rescue” drugs such as fosfomycin should be increased for critically ill patients.

Infection control teams play a definitive role in antibiotic AMS programs by assisting with prompt detection of MDROs and promoting compliance with standard and transmission-based precautions. They also facilitate the use of other infection prevention strategies, such as implementing care bundles to prevent hospital-acquired infections, ensuring hand hygiene compliance and educating not only staff and patients, but also visitors about infection prevention topics. Therefore, infection control teams play a crucial part in AMS. Infection control and epidemiology professionals launched the concept of “synergy” to highlight the joint effort of infection prevention and AMS [65]. This initiative has three key aims: (1) minimize nosocomial infections, (2) decrease the use of additional antibiotics and (3) reduce MDROs.

## Current and new treatment options

Two major organizations, the IDSA and ECDC, have analyzed the repertoire of drugs in the antibiotic R&D pipeline, and there are very few offering significant benefits over existing therapies (Table 1) [66].

**Table 1:** Adapted from [66]

Company	Product	Class/structure	Mechanism of action	Lead indication
Actelion Ltd. (SIX:ATLN)	Cadazolid (179811)	Oxazolidinone–quinolone hybrid	Inhibit DNA replication and protein synthesis	*CDAD*
Affinium Pharmaceuticals Ltd.	AFN-1252	FabI enoyl-(acyl carrier protein) reductase inhibitor	Inhibit fabI	ABSSSI
CrystalGenomics Inc. (KOSDAQ:83790)	CG400549	FabI enoyl-(acyl carrier protein) reductase inhibitor	Inhibit fabI	Complicated ABSSSI
GlaxoSmithKline plc(LSE:GSK; NYSE:GSK)	GSK1322322	Peptide deformylase (PDF) inhibitor	Inhibit PDF	Bacterial infection
Furiex Pharmaceuticals Inc. (NASDAQ:FURX)/Johnson & Johnson (NYSE:JNJ)	JNJ-32729463 (JNJ-Q2)	Fluoroquinolone	Inhibit DNA replication	ABSSSI, CAP
Nabriva Therapeutics AG/Forest Laboratories Inc. (NYSE:FRX)	BC-3781	Pleuromutilin	Inhibit protein synthesis	ABSSSI
Novartis AG (NYSE:NVS)	LFF571	Thiopeptide	Inhibit protein synthesis	*CDAD*
PolyMedix Inc. (OTCBB:PYMX)	Brilacidin (PMX-30063)	Defensin mimetics	Membrane lysis	ABSSSI
Rib-X Pharmaceuticals Inc.	Radezolid (RX-1741)	Oxazolidinone	Inhibit protein synthesis	CAP
Tetraphase Pharmaceuticals Inc.	Eravacycline (TP-434)	Tetracycline	Inhibit protein synthesis	cIAI
Theravance Inc. (NASDAQ:THRX)	TD-1792	Beta-lactam-glycopeptide hybrid	Inhibit cell wall synthesis	Gram-positive cSSSI
Wockhardt Ltd. (BSE:532300)	WCK 771	Fluoroquinolone	Inhibit DNA replication	*S. aureus*
Wockhardt Ltd. (BSE:532300)	WCK 2349	Fluoroquinolone	Inhibit DNA replication	Gram-positive cSSSI
Nanotherapeutics Inc.	Ramoplanin	Lipoglycodepsipeptide	Inhibit cell wall production	CDAD
2 M BioTech L.P.	CBR-2092	Rifamycin–quinolone hybrid	Inhibit DNA replication and RNA synthesis	*S. aureus*
Achaogen Inc.	ACHN-975	N-acetylglucosamine deacetylase (LpxC) inhibitor	Inhibit LpxC	Gram-negative
aRigen Pharmaceuticals Inc./Green Cross Corp.	WAP-8294A2	Depsipeptide	Disrupt membrane	MRSA
Basilea Pharmaceutica AG (SIX:BSLN)	BAL30072	Beta-lactam (monocyclic)	Inhibit cell wall synthesis	Gram-negative
FAB Pharma S.A.S.	FAB001 (MUT056399)	FabI enoyl-(acyl carrier protein) reductase inhibitor	Inhibit fabI	*S. aureus*
Kalidex Pharmaceuticals Inc.	KPI-10	Fluoroquinolone	Inhibit DNA replication	Gram-negative and Gram-positive
Kyorin Pharmaceutical Co. Ltd. (Tokyo:4569)	KRP-AM1977X	Quinolone	Inhibit DNA replication	Gram-positive
MicuRx Pharmaceuticals Inc.	MRX-I	Oxazolidinone	Inhibit protein synthesis	Gram-positive
Novacta Biosystems Ltd.	NVB302	Lantibiotic	Inhibit cell wall synthesis	*CDAD*
Rempex Pharmaceuticals Inc.	Carbavance (RPX7009/biapenem)	Beta-lactamase inhibitor with boron core (RPX7009); beta-lactam (biapenem)	Inhibit cell wall synthesis	Gram-negative
Shionogi & Co. Ltd.(Tokyo:4507)/GlaxoSmithKline plc (LSE:GSK; NYSE:GSK)	S-649266	Beta-lactam	Inhibit cell wall synthesis	CAP
Tetraphase Pharmaceuticals Inc.	TP-2758	Tetracycline	Inhibit protein synthesis	cUTI

MRSA is a major MDRO in ICU’s around the globe. It is frequently treated with vancomycin, linezolid, daptomycin, ceftaroline or tigecycline. Linezolid was found to be superior for the treatment of HAP in the ZEPHyR trial [67] in terms of cure rate and nephrotoxicity. Daptomycin is a good alternative, but not licensed for pneumonia as the drug is inactivated by surfactant [68]. As a new alternative for MRSA, the novel tedizolid was found to be non-inferior to linezolid in early clinical response for an acute bacterial skin and skin structure infections (ABSSSI) [69]. Moreover, recent in vitro studies also confirmed activity against all Gram-positive isolates, which were inhibited by ≤1 μg/mL tedizolid in samples of patients with HAP. Antimicrobial susceptibility testing revealed that the MIC_90_ of tedizolid was 0.5 μg/mL for MRSA, which was fourfold lower than that of linezolid [70].

*Enterococci* are Gram-positive facultative anaerobes that live as commensals in the gastrointestinal tract, but they are not as virulent as *S. aureus* or *S. pneumoniae*. Donskey et al. [71] conducted a prospective study and found that vancomycin-resistant enterococci (VRE) in stool were associated with treatment with anti-anaerobic antimicrobials. More recently, an animal study found that in contrast to vancomycin, fidaxomicin (a new class of narrow spectrum macrocyclic antibiotic drugs) treatment caused minimal disruption of the intestinal flora and did not increase the rate of VRE and extended-spectrum β-lactamase-producing Klebsiella pneumoniae (ESBL-Kp) colonization [72]. Limiting the use of such agents may help decrease the spread of VRE. *Enterococci* spp. are likely to function as a reservoir of drug resistance determinants and can serve as the springboard for the spread of these genes to other Gram-positive pathogens [73]. It is important to highlight that there is a relatively high rate of VRE not susceptible to linezolid observed in ICU patients [31]. New agents like dalbavancin, oritavancin and telavancin seem promising [74].

Treatment of carbapenem-resistant *Enterobacteriaceae* spp. using monotherapy is often inadequate, and challenging cases may require the use of highly toxic agents. Aminoglycosides, polymyxins, tigecycline, fosfomycin and occasionally fluoroquinolones form the backbone of treatment. Recently, the US Food and Drug Administration (FDA) issued a warning regarding the risk of increased mortality in patients treated with tigecycline, observed in the clinical trials [75]. An observational study by Montravers et al. [76] found it can be used safely even in severe infections. However, a debate about the potential benefit of a high dosage regimen (200 mg daily) still needs to be resolved. Combination antimicrobial therapy has been shown to be superior in terms of cure rate when compared to monotherapy in multiple observational studies [77]. Emerging treatment options include ceftazidime–avibactam. Avibactam is a novel β-lactamase inhibitor with activity against class A, class C and some class D β-lactamases. It has activity against KPC-type carbapenemases and some OXA enzymes; however, it has no activity against metallo-β-lactamases (such as NDM-1) [78]. Ceftazidime–avibactam was approved by the Food and Drug Administration (FDA) in 2015 for the treatment of complicated intra-abdominal infections and complicated UTIs [79]. A newly released fifth-generation cephalosporin ceftolozane combined with a beta-lactamase inhibitor tazobactam has potent activity against *P. aeruginosa*, including various MDR types [80]. Another alternative for the treatment of MDR, XDR and PDR *Pseudomonas* ssp. is the use of intra-tracheal antibiotics (ITA), consisting mainly of aminoglycosides and polymyxins, with encouraging results seen to date. In mechanically ventilated patients, the use of ITA for prevention or treatment of VAP has shown less toxicity as compared to systemic formulations [81]. A recent international survey reported that intra-tracheal antibiotic administration is a common therapeutic modality in ICUs; however, practice varies widely [82]. *Acinetobacter* spp. similar to *P. aeruginosa* have ample ways to develop and horizontally transfer resistance. Of greatest significance is the production of beta-lactamases and aminoglycoside-modifying enzymes [18]. Combination therapy including a polymyxin has commonly been the mainstay of treatment for *Acinetobacter spp*. However, a recent paper has highlighted the risk of nephrotoxicity when colistin and vancomycin are used in combination with no benefit to clinical outcome [83].

More recently, studies have been conducted revealing the benefits of treatment with “new” combination therapies for XDR or PDR organisms. In terms of PDR Gram-negative infections, Bulik et al. [84] found that double-carbapenem therapy (ertapenem and doripenem) for carbapenemase-producing *K. pneumoniae* had an enhanced efficacy over either agent alone both in vitro and in vivo in animal models. Giamarellou et al. [85] found that the use of ertapenem plus doripenem or meropenem resulted in treatment success without relapse at follow-up in patients with PDR *K. pneumoniae.*

As the vast majority of the antibiotic treatment is short term, many companies struggle to generate profit from the sales of anti-infective agents. The majority of products being approved are second-, third- or fourth-generation antibiotics, meaning they are follow-up compounds, without a novel mechanism of action. In addition to this, many novel antibiotics have struggled to reach the market due to difficulties in demonstrating efficacy or unacceptable side effects. Furthermore, for licensing purposes, new antibacterials must demonstrate non-inferiority against already marketed drugs. Finally, healthcare authorities are frequently reluctant to pay the high costs associated with clinical trials, hindering the development of novel antimicrobial agents.

As a result of the declining effectiveness of existing antibiotics and the steady decrease in new antibiotic development, alternative approaches to antibiotic therapy are appearing including immunotherapy and the use of bacteriophages. Immunotherapy initiated at the outset of an antibiotic course has the potential to result in beneficial effects; however, use is frequently limited by a narrow spectrum of action against specific target antigens. Another attractive approach to address the critical need for new antibiotics is the engineering of various components of the immune response such as monoclonal antibodies. For instance, a randomized, placebo-controlled phase I study found that IC43 (recombinant outer membrane protein-based vaccine against *P. aeruginosa*) could induce an antibody response in healthy volunteers [86]. Phase II and III studies are being conducted in mechanically ventilated patients (ClinicalTrials.gov Identifier: NCT01563263). Another cutting edge approach is the use of bacteriophages or phages. These are bacterial viruses that invade bacterial cells, disrupt metabolism, cause bacteriolysis and are highly specific and effective [87].

## Future prospects

Infections with MDROs are already a threat in a number of countries. It is expected that in others with existing low level of MDROs, the number of infections due to MDR, XDR and even PDR organisms will rise. These infections are associated with an increased consumption of healthcare resources manifested by a prolonged hospital stay and an augmented mortality. In order to reduce antibiotic resistance rates, a strategy minimizing the use of broad-spectrum antibiotics and ensuring prompt antibiotic administration should be adopted. The development of rapid diagnostic tests will help by both shortening duration of therapy and allowing prompt-targeted therapy. The implementation of more accessible therapeutic drug monitoring will help to optimize drug administration and enable a more personalized approach to treatment. Some points require further investigation in clinical trials, such as the heterogeneity of patients admitted to ICU and the need for new drug development.

Since late 1980s, no new class of antibiotics has been discovered that is available for treatment of systemic bacterial infections. This discovery void is still impacting the therapeutic options to treat infections caused by multidrug- or extensively drug-resistant bacteria. Recent findings regarding existing antibiotics combinations should also be mentioned. Combination therapy regimens with daptomycin with either oxacillin (enhancing in vivo efficacy to daptomycin monotherapy due to a daptomycin–oxacillin seesaw phenomenon in vitro) [88] or β-lactams [89] have been showed to enhance daptomycin efficacy against daptomycin-resistant strains. These examples just want to illustrate a “think outside of the box” model for existing drug combination for new research in the future. Certainly, it would need a deep remodel of the current drug regulation for new antibacterials and to adequately stimulate investment by the industry for new antimicrobial developments and ultimately to non-antibiotic approaches.

In summary, a steady resistance increase particularly to Gram-negatives, rising minimum inhibitory concentration in methicillin-resistant *Staphylococcus aureus* and the spread of multiresistant strains of pathogens in patients without classic risk factors in the ICU are key areas to delineate in future studies. Guidelines and recommendations will need to incorporate the ecology of the hospital setting and the severity of patient illness to provide a personalized patient approach to antimicrobial treatment in the future.
